# Neuroendocrine mechanisms that connect feeding behavior and stress

**DOI:** 10.3389/fnins.2014.00312

**Published:** 2014-10-01

**Authors:** Zane B. Andrews, Alfonso Abizaid

**Affiliations:** ^1^Physiology, Monash UniversityCalyton, VIC, Australia; ^2^Neuroscience, Carleton UniversityOttawa, ON, Canada

**Keywords:** stress, psychological, food intake, dopamine, ghrelin, leptin, depression, obesity

Research during the past decade highlights the strong link between appetitive feeding behavior, reward, and motivation. Interestingly, stress levels can affect feeding behavior by manipulating hypothalamic circuits and brain dopaminergic reward pathways. Indeed, animals and people will increase or decrease their feeding responses when stressed. In many cases, acute stress leads to a decrease in food intake, yet chronic social stressors are associated to increases in caloric intake and adiposity. Interestingly, mood disorders and the treatments used to manage these disorders are also associated with changes in appetite and body weight. These data suggest a strong interaction between the systems that regulate feeding and metabolism and those that regulate stress and ultimately mood. This Research Topic compiles a number of review and research articles that focus how hormonal mechanisms regulate the nexus between feeding behavior and stress. It highlights the hormonal regulation of hypothalamic circuits and/or brain dopaminergic systems, as the potential sites controlling the converging pathways between feeding behavior and stress.

The regulation of energy balance is controlled by hypothalamic nuclei that include the arcuate nucleus (ARC), a mediobasal hypothalamic region that has access to circulating peripheral signals including those that regulate metabolism and stress. Neuropeptide Y (NPY) neurons within the ARC were identified over three decades ago as potent stimulators of food intake and adiposity, and later on as important regulators of the hypothalamic-pituitary-adrenal (HPA) axis, sympathetic activity, and anxiety related behaviors. In this issue, Sundstrom et al. (2013) use a Zebrafish model to illustrate the specific affinities for NPY peptides with a number of specific Y-family receptors. Their results clearly suggest that NPY and its related peptides, all of which are released in response to stress, bind preferentially to the brain and other tissues like heart and kidney that are affected by stressors. Given that NPY cells in the ARC are extremely sensitive to circulating hormones, it is not surprising that these hormones modulate appetitive behaviors. The review by Keen-Rhinehart (Keen-Rhinehart et al., 2013) focuses on the neuroendocrine roles of ghrelin and leptin in appetitive behavior, and provides an excellent overview of the neuroendocrine regulation of appetitive behavior, which includes not only food intake but also foraging, hoarding, chewing, and swallowing. Patton and Mistlberger (2013) provide an excellent review on how neuroendocrine mechanisms influence circadian meal timing. They show how food availability entrains food anticipation and critically examine the role of peripheral metabolic hormones as potential internal stimuli entraining behavioral and physiological rhythms. Key hormones discussed are corticosterone, ghrelin, leptin, insulin, glucagon, and glucagon-like peptide (GLP1). The influence of metabolic hormones on metabolism in relation to timing events is also highlighted by Cahill et al. (2013) showing that circannual changes in stress and feeding hormones affect food seeking behaviors. This review also highlights the importance of circulating levels of glucocorticoids, ghrelin, and leptin to the fluctuations in feeding-related behaviors associated with seasonality, and discuss in detail how metabolic stressors like restricted food availability influence appetitive and cosummatory eating behavior. In addition to nutritional stressors, psychological stressors can have both organizational and activational effects on the brain systems controlling food intake and energy balance. For instance, Spencer (2013) addresses how early-life nutrition, stress, and hormonal profiles influence the development of the HPA axis, as well as the development of the hypothalamic circuitry that regulates feeding behavior. The paper by Patterson and Abizaid (2013) discusses how psychological stress, and in particular social stress, represents also a metabolic challenge that is associated with hormonal changes akin to those observed in animals that are calorically restricted. Among these, ghrelin, a gut peptide that stimulates appetite and promotes adiposity, is released during stress and is a factor that contributes to stress induced caloric intake and weight gain. Furthermore, Patterson et al. (2013) provide data indicating the intricacy of the PVN in mediating the effects of ghrelin on stress-induced caloric intake and weight gain. Uchida et al. (2013) provide an excellent description detailing the use of genetically-modified mouse models to understand the effects of ghrelin on feeding behavior. Specifically, they show that ghrelin receptor signaling on catecholaminergic neurons influences chronic stress-induced food reward. This is followed by Schellekens, Dinan, and Cryan's review illustrating how ghrelin receptor dimerization influences stress and reward (Schellekens et al., 2013). This new dimension in ghrelin receptor signaling shows that independent from ghrelin binding, ghrelin receptors interact with serotonin and dopamine receptors to influence aminergic tone and modulate stress and reward processes. In support of this, Wellman et al. (2013) discuss the importance of ghrelin receptor signaling on modulating psychostimulant action, including the action of addictive drugs like nicotine, cocaine, and amphetamine. This review shows that ghrelin universally enhances sensitivity to psychostimulants and that ghrelin receptor antagonism may be an important therapeutic target to help quit smoking and curb addictive behaviors. Ghrelin may also be implicated in the narcotic drug seeking following stress, given that, as shown by Sedki et al. (2013), blocking CRF receptors or adrenalectomy do not attenuate the effects of food restriction on heroin seeking behavior.

In addition to ghrelin, other peptide hormones have been implicated in stress induced feeding and psychopathology. For instance, Merali and colleagues draw attention to the bombesin family of peptides and provide intriguing evidence that they regulate stress-induced anorexia or obesity depending on subtle differences in neural circuitry and neurochemical phenotypes (Merali et al., 2013). Skibicka reviews the central actions of GLP1 of food and drug reward. In this review, we learn that GLP1 acts in the mesolimbic dopamine system to reduce hunger driven food intake, the hedonic value of food and food motivation (Skibicka, 2013). Emerging evidence shows that GLP1 will reduce not only food-based reward behavior but also cocaine, amphetamine, and alcohol rewards. Finally, a paper by McQuaid et al. and a commentary by Tabak both highlight the role of oxytocin receptor function and how it links early-life stress and vulnerability to depression (McQuaid et al., 2013; Tabak, 2013). They show that human gene polymorphisms in the oxytocin receptor affect depressive symptoms when exposed to early life maltreatment. Not surprisingly, this collection of manuscripts provide for new links between unconventional metabolic hormones and pathological conditions associated with continued exposure to stress. This is further highlighted Hryhorczuk and colleagues, who examine how metabolic disturbances connect obesity and depression. In particular, these authors highlight evidence showing that the metabolic and hormonal changes that accompany obesity have a direct and indirect impact on mood and emotion (Hryhorczuk et al., 2013).

Collectively, the articles published in this research topic offer an in depth analysis of how neuroendocrine mechanisms connect feeding behavior and stress. All articles offer unique and novel elements on this topic and we hope you enjoy.

## Conflict of interest statement

The authors declare that the research was conducted in the absence of any commercial or financial relationships that could be construed as a potential conflict of interest.
